# Genetic differentiation of *Rubus chamaemorus* populations in the Czech Republic and Norway after the last glacial period

**DOI:** 10.1002/ece3.4101

**Published:** 2018-05-02

**Authors:** Leona Leišová‐Svobodová, Jade Phillips, Inger Martinussen, Vojtěch Holubec

**Affiliations:** ^1^ Crop Research Institute Prague Czech Republic; ^2^ School of Biosciences University of Birmingham Birmingham UK; ^3^ Norwegian Institute of Bioeconomy Research Holt Norway

**Keywords:** cloudberry populations, conservation, genetic diversity, Krkonose Mountains, microsatellites, multivariate data analysis

## Abstract

The population structure of cloudberry (*Rubus chamaemorus* L.)*,* collected from Krkonose Mountains (the Czech Republic), continental Norway and Spitsbergen, was examined using microsatellite analyses (SSR). Among 184 individuals, 162 different genotypes were identified. The overall unbiased gene diversity was high ($\hat{h}=0.463$). A high level of genetic differentiation among populations (*F*_ST_ = 0.45; *p* < .01) indicated restricted gene flow between populations. Using a Bayesian approach, six clusters were found which represented the genetic structure of the studied cloudberry populations. The value of correlation index between genetic and geographical distances (*r* = .44) indicates that gene flow, even over a long distance, could exist. An exact test of population differentiation showed that *Rubus chamaemorus* populations from regions (Krkonose Mountains, continental Norway and Spitsbergen) are differentiated although some individuals within populations share common alleles even among regions. These results were confirmed by AMOVA, where the highest level of diversity was found within populations (70.8%). There was no difference between 87 pairs of populations (18.7%) mostly within cloudberry populations from continental Norway and from Spitsbergen. Based on obtained results, it is possible to conclude that Czech and Norwegian cloudberry populations are undergoing differentiation, which preserves unique allele compositions most likely from original populations during the last glaciation period. This knowledge will be important for the creation and continuation of in situ and ex situ conservation of cloudberry populations within these areas.

## 1 INTRODUCTION

**Figure 1:**
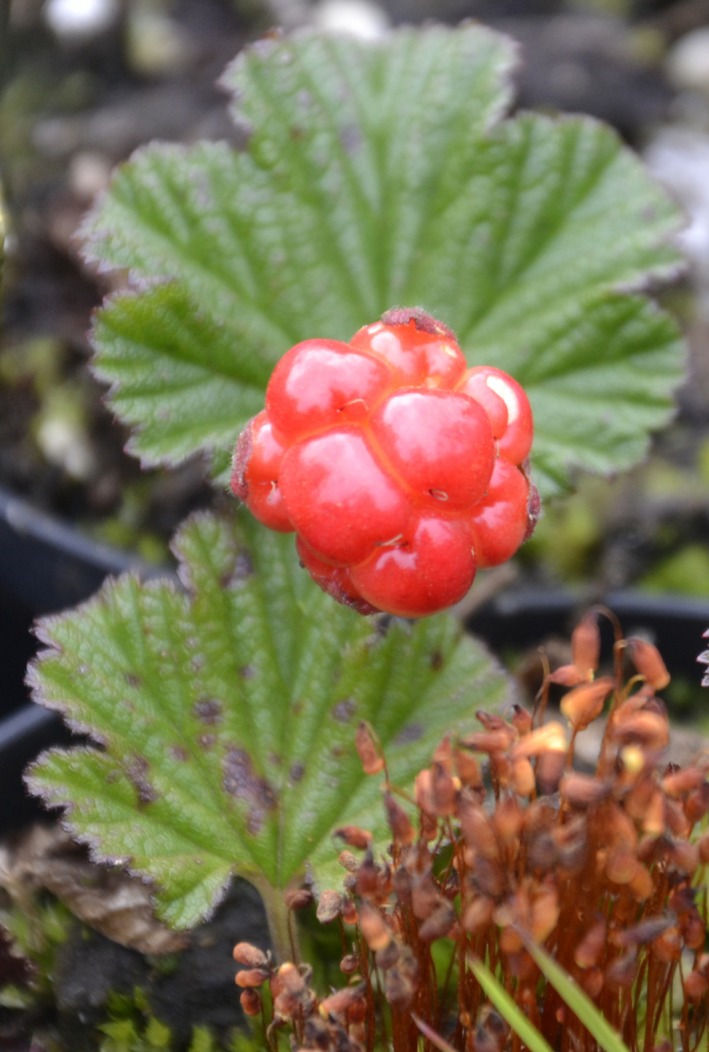
Photo of cloudberry (*Rubus chamaemorus*) with fruit (V. Holubec)

Cloudberry (*Rubus chamaemorus* L. Sp. Pl. 494, 1753; Family: *Rosaceae*; Figure 1) is a perennial dioecious plant with boreal circumpolar distribution. It is an octoploid plant with 2C = 2*n* = 8*x* = 56 with the estimated genome size 2.46 pg/2C (Thiem & Sliwinska, 2003), which is about 3.8 Gbps. The species mostly reproduces asexually and spreads locally using an extensive rhizomatic system (Taylor, 1971). Sexual reproduction is also important, although rare, as the fruits are edible and of economic value especially in Scandinavia. They are tasty and contain multiple compounds beneficial to human health, particularly vitamins, minerals, and antioxidants (tannins, flavone, quercetin, and naringenin). In particular, the Alaskan Inuit and the Norwegian Sami use cloudberry as an important contribution to their diet (reviewed by Nilsen, 2005).

The opinions on cloudberry population diversity are not clear. Korpelainen, Antonius‐Klemola, and Werlemark (1999) published the results of a diversity study of three Norwegian populations based on RAPD, SSR, and hybridization methods. Although cloudberry expressed clear variation in morphology, the level of genetic variability appeared to be low. This is also true for detected allozyme variability of the same cloudberry populations (Korpelainen, 1994).

Debnath (2007) used intersimple sequence repeat (ISSR) PCR analysis to study genetic variability of 48 cloudberry clones from four Canadian Provinces. They found a substantial degree of genetic diversity, but only 8% of the total variation could be explained by geographical distribution (Debnath, 2007).

Interesting results were gained by Ehrich, Alsos, and Brochmann (2008) who studied 45 cloudberry populations through their main distribution area and two populations from Scotland. Based on AFLP analysis, they found a high level of genetic diversity among all populations, and more than one clone was found in nearly every local population. The phylogeographical pattern was assessed to be shallow. The authors concluded that the present circumpolar cloudberry distribution area has been colonized at least twice and possibly several times. The highest level of genetic diversity was found in the Taimyr Peninsula, Russia (Ehrich et al., 2008).

As a glacial relic, *Rubus chamaemorus* occurs in Alaska, British Columbia, SW Greenland, Siberia, Kamchatka, Kuril Islands, Sakhalin, North Korea, Scotland, and Poland (Hultén, 1968). The species also occurs in the Czech Republic (CR; Holub, 1995; Taylor, 1971), where it is the southernmost distribution in Europe. Here, it is recognized as a critically endangered species (Grulich, 2012) and is only found in two localities; both in the Krkonose Mountains (Kubát, 2002). There is no information about the diversity of these populations and whether these populations have genetically diverged from populations of the closest main distribution area, such as the Scandinavian region. The results of such a study would be very useful for conservation management of cloudberry in the Czech Republic or in other places of its marginal occurrence.

Therefore, the main aim of this study was to assess the genetic diversity, differentiation, and structure of isolated populations of *Rubus chamaemorus* from the Czech Republic with the comparison of cloudberry populations collected throughout Norway from the south coast to Spitsbergen. These results were then used to address the following questions: (1) Has the differentiation of populations of *Rubus chamaemorus* already occurred from the Czech Republic, continental Norway, and Spitsbergen after last glacial period? (2) Is there any gene flow among populations? (3) How much genetic diversity is maintained in these naturally fragmented populations of *Rubus chamaemorus* and what does this mean for its conservation management?

## 2 MATERIALS AND METHODS

### 2.1 Population sampling and DNA extraction

**Figure 2:**
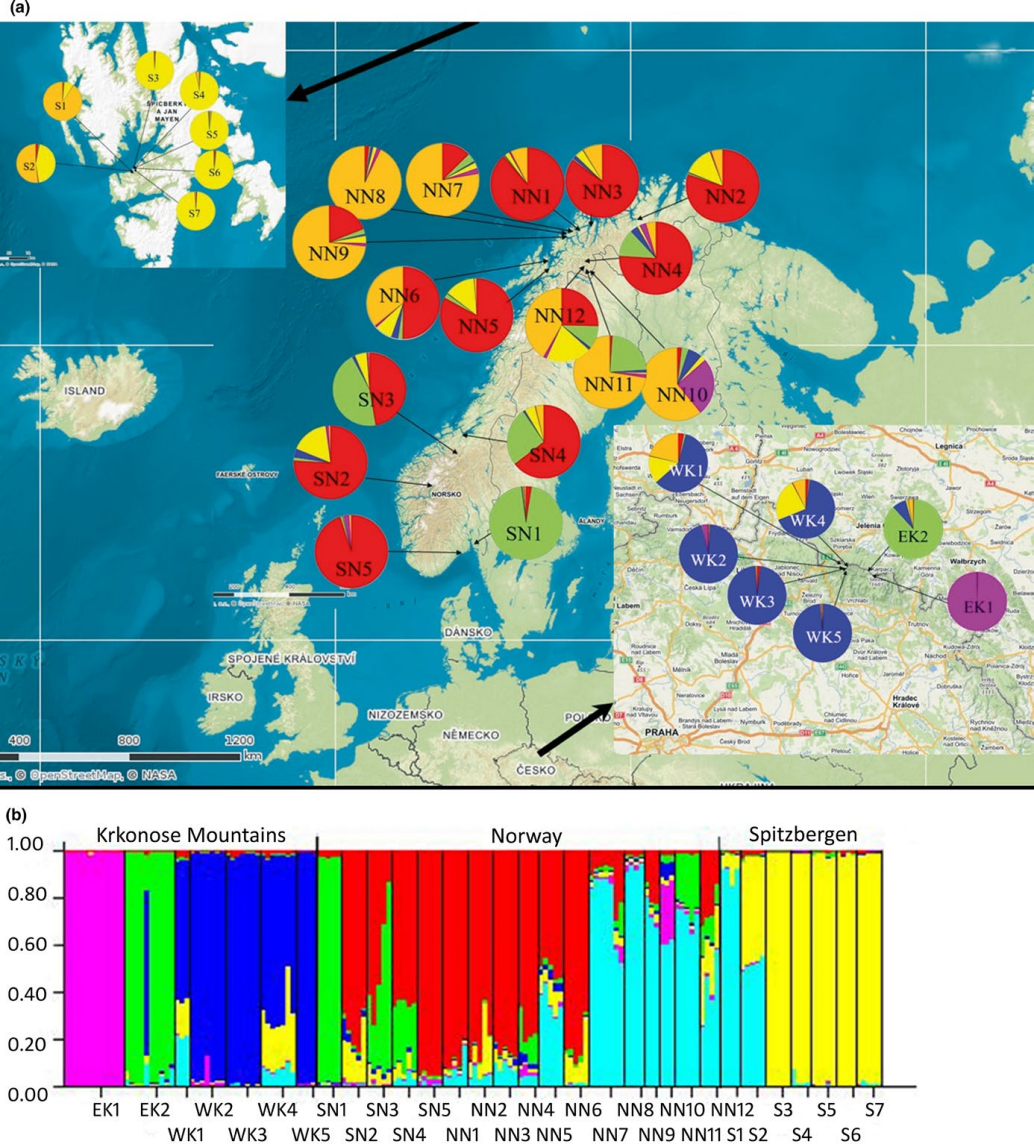
(a) Sample localities of *Rubus chamaemorus* populations with pie charts describing the proportions of individuals classified into one of the six clusters defined using the Bayesian approach (Pritchard et al., 2000). Each color represents one of six clusters. (b) Direct output from Structure software for all populations for *K* = 6

Thirty‐one populations of *Rubus chamaemorus*, including 184 individuals, were sampled in 2015 and 2016 (Table 1; Appendix S1). Sixty seven samples were collected in CR in the Krkonose Mountains, 117 samples in Norway including 36 samples from Spitsbergen (Figure 2a). The sampling area ranged from 6°E to 23°E and from 50°N to 78°N. Except from Krkonose Mountains where all supposed ramets were collected, five samples were taken from each locality. Fresh leaves were dried (Staats et al., 2011) and stored in ziplock plastic bags with silica gel until DNA extraction. Genomic DNA was extracted from silica gel dried material using a CTAB protocol (Doyle & Doyle, 1987; Drabkova, Kirschner, & Vlček, 2002), and the quality of the extracted DNA was checked on 0.7% agarose gels.

**Table 1:** Characteristics of 31 *Rubus chamaemorus* local populations and their diversity evaluation based on 28 SSR loci analysis

Population	*n* a	*N* b	Country	Region	Locality	Altitude mnm	Latitude N	Longitude E	Collection date^c^	$\hat{h}$ d	% *P* e	*I* f
EK1	13	12	CZ	East Krkonose Mountains	Upske raseliniste	1,427	50°44′13.7″	15°42′40.7″	21.07.2015	0.289	58.3	0.424
EK2	17	10	CZ		Certova louka	1,404	50°44′49.7″	15°40′49.9″	21.07.2015	0.319	66.7	0.523
WK1	8	3	CZ	West Krkonose Mountains	Hranicni louka	1,244	50°47′16.1″	15°30′4.7″	21.07.2015	0.304	50.0	0.371
WK2	8	7	CZ		Mumlavska louka	1,329	50°45′55.1″	15°32′5.4″	21.07.2015	0.291	57.7	0.424
WK3	7	7	CZ		Pancavska louka	1,320	50°45′53.3″	15°32′17.8″	21.07.2015	0.272	56.0	0.385
WK4	9	7	CZ		Labska louka	1,362	50°46′19.8″	15°32′20.5″	21.07.2015	0.316	66.7	0.441
WK5	5	4	CZ		Harrachova louka	1,380	50°45′26.2″	15°32′5.3″E	21.07.2015	0.273	47.8	0.339
SN1	5	5	NO	South Norway	Fredrikstad (3)	27	59°01′45.5″	11°01′20.2″	01.08.2016*	0.351	65.4	0.479
SN2	5	5	NO		Ænes and Odda (11)	110	60°04′36″	6°07′14.8″	01.08.2016*	0.362	70.4	0.525
SN3	5	5	NO		Kongsvoll protected area (13)	913	62°18′5.8″	9°36′41.6″	01.08.2016*	0.353	70.4	0.556
SN4	5	5	NO		Trondheim (14)	221	63°23′49.7″	10°14′10″	01.08.2016*	0.370	76.9	0.518
SN5	5	5	NO		Tønsberg on mainland (18)	5	59°11′1″	10°28′53″	01.08.2016*	0.332	59.3	0.424
NN1	5	5	NO	North Norway	Tromso (21)	104	69°39′22.9″	18°55′57.4″	01.08.2016*	0.366	66.7	0.538
NN2	5	5	NO		Alta (22)	127	69°54′26.5″	23°28′30.8″	10.07.2015*	0.428	76.0	0.654
NN3	5	5	NO		Svensby (23)	8	69°46′32″	19°51′38.1″	12.07.2015*	0.400	70.4	0.542
NN4	4	4	NO		Dividalen_holt on road 87 (24)	298	68°43′11.8″	19°45′14″	15.07.2015*	0.450	80.0	0.662
NN5	5	5	NO		Narvik (25)	84	68°37′30.6″	16°36′19.8″	16.07.2015*	0.390	70.4	0.582
NN6	5	5	NO		Harstad (26)	130	68°47′22.5″	16°29′3″	17.07.2015*	0.335	61.5	0.499
NN7	7	7	NO		Kvaloya island (R1)	157	69°39′4.4″	18°29′27.7″	28.07.2016	0.424	85.2	0.669
NN8	4	4	NO		Kvaloya island, coast (R2)	15	69°37′42.1″	18°8′7.2″	28.07.2016	0.233	46.4	0.312
NN9	3	3	NO		Kvaloya island, coast (R3)	13	69°35′29.7″	18°2′37.6″	28.07.2016	0.356	61.5	0.475
NN10	3	3	NO		Dividalen (R7)	513	68°41′16.9″	19°48′4.2″	30.07.2016	0.290	46.2	0.342
NN11	5	5	NO		Dividalen (R8)	500	68°41′18.8″	19°47′55.7″	30.07.2016	0.318	57.7	0.447
NN12	5	4	NO		Dividalen (R9)	500	68°42′57.0″	19°46′06.3″	30.07.2016	0.317	55.6	0.424
S1	6	4	NO	Spitsbergen	Colesdalen (R11)	57	78°6′36.2″	15°3′54.3″	01.08.2016	0.235	42.3	0.299
S2	5	5	NO		Colesdalen (R12)	59	78°6′37.1″	15°3′52.4″	01.08.2016	0.234	42.3	0.312
S3	5	5	NO		Colesbuchta (R13)	13	78°7′45″	14°59′33.9″	01.08.2016	0.267	42.3	0.346
S4	5	4	NO		Colesbuchta (R14)	12	78°7′44.7″	14°59′34.1″	01.08.2016	0.213	34.6	0.271
S5	5	5	NO		Colesbuchta (R15)	14	78°7′46.1″	14°59′31″	01.08.2016	0.273	50.0	0.388
S6	5	4	NO		Colesbuchta (R16)	16	78°7′46.5″	14°59′29.7″	01.08.2016	0.214	42.3	0.286
S7	5	5	NO		Colesbuchta (R17)	65	78°7′49.7″	15°0′5.6″	01.08.2016	0.194	34.6	0.280
Total	184	162								**0.463**	**96.4**	**0.937**
										0.315	58.4	0.443

*Samples were collected by Jade Phillips.

a Number of plants analyzed.

b Number of distinct genotypes identified.

c Samples were collected by Jade Phillips.

d Nei′s (1978) unbiased heterozygosity.

e Percentage of polymorphic loci.

f Shannon information index as a measure of gene diversity (Shannon & Weaver, 1949).

### 2.2 Microsatellite analysis

To study *Rubus chamaemorus* populations, a set of 24 microsatellite loci were chosen from those reported by Graham, Smith, Woodhead, and Russell (2002), Graham et al. (2004) and by Castillo, Reed, Graham, Fernández‐Fernández, and Bassil (2010). The PCRs with fluorescently labeled primers (6‐fam, vic, ned. and pet) were performed in a reaction volume of 15 μl, which consisted of a 1× Mg‐free buffer (Biotools, Spain), 2 mmol/L MgCl_2_, 0.33 mmol/L of each dNTP (Invitrogen, Germany), 0.33 μmol/L of each primer (Generi Biotech, the Czech Republic), 1U Tth polymerase (Biotools, Spain), and 50 ng DNA template. The PCR was performed in a Sensoquest Labcycler (Goettingen, Germany) under the following conditions: an initial denaturing step of 95°C for 5 min, followed by 35 cycles of 30 s at 95°C, 30 s at primer pair‐specific annealing temperature and then 40 s at 72°C, and finally finished at 72°C for 5 min. The analysis of the PCR products was performed using capillary electrophoresis on the sequencer ABI PRISM 3130 (Applied Biosystems, the United States). A multiplexed configuration of four reactions was used in one analysis. The internal size standard LIZ500 (Applied Biosystems, the United States) was used. The electropherograms were processed using the GeneMapper software (Applied Biosystems, the United States).

### 2.3 Data analysis

Clone identity was determined using multilocus matches for codominant data. The probability of identity (i.e., estimating the probability of randomly matching two unrelated (PI) or related (PIsib) individuals by a particular set of loci) were calculated based on the distribution of allele frequencies in population samples using software GENECAP (Wilberg & Dreher, 2004).

A matrix of distances between all of the samples was calculated using the simple matching dissimilarity coefficient in the DARwin software (http://darwin.cirad.fr/darwin; Perrier & Jacquemoud‐Collet, 2006). For clustering, an unweighted Neighbour‐joining method (UNJ) was used as its cophenetic coefficient *r* showed the highest value (0.943). The support for the phenogram branches was obtained using 2,000 bootstrap resamplings.

The diversity statistics for each population included the percentage of polymorphic loci, the average diversity of the loci using Nei's unbiased gene diversity $\hat{h}$ (Nei, 1973), and the Shannon information index (Lewontin, 1972; Shannon & Weaver, 1949). All of these statistics were calculated using the POPGENE software, version 1.32 (Yeh, Boyle, Rongcai, Ye, & Xiyan, 1999).

The divergence statistics were estimated using the hierarchical analysis of molecular variance (AMOVA; Excoffier, Smouse, & Quatro, 1992) which was performed using Arlequin version 3.5 (Excoffier & Lischer, 2010). It was used to partition the total genetic variation into three specific hierarchical levels: among the genotypes collected within localities, among the different localities within three “regions” (CR, continental Norway, and Spitsbergen), and between the “regions.” The significance levels for the resultant molecular variance components were computed by default 1,023 nonparametric permutation procedures (Excoffier et al., 1992). The degree of population subdivision was measured by Wright's fixation index (*F*_ST_). Arlequin software was also used to evaluate the correlation between a matrix of logarithmic geographical distances and a matrix of *F*_ST_ values using a Mantel test with the recommended 10,000 permutations.

An exact test for population differentiation was calculated using the Tools for Population Genetic Analyses (TFPGA; version 1.3; Miller, 1997) with recommended 100,000 permutation steps.

Another approach to studying the population structure analysis is based on Bayesian statistics. Structure version 2.3.4 (Pritchard, Stephens, & Donnelly, 2000) was used to determine the genetic architecture of the *Rubus chamaemorus* populations. Ten independent runs of one–20 groups (*K *=* *1–20) were performed using locprior model with admixture and correlated allele frequency (Falush, Stephens, & Pritchard, 2003; Hubisz, Falush, Stephens, & Pritchard, 2009) with the recommended 20,000 Markov chain iterations after a burning period of 10,000 iterations. The optimal value of *K* was estimated based on ln (*K*) and on the Δ*K* calculation, which considers the rate of change in the ln *P(D)* values among successive *K* runs to account for patterns of dispersal that are not homogeneous among populations (Evanno, Regnaut, & Goudet, 2005). The number (*K*) of clusters into which the sample data (*X*) were fitted with posterior probability Pr (*X|K*) was estimated using the same model with 1,000,000 Markov chain iterations after a burning period of 100,000 iterations (Evanno et al., 2005).

## 3 RESULTS

A total of 180 alleles in 28 microsatellite loci were detected when analyzing 184 *Rubus chamaemorus* samples with 24 primer pairs. We identified a total of 162 multilocus genotypes. Forty matches were found with PI_sib_ < 0.05; therefore, they were excluded from further analyses (Table 1).

The number of alleles per locus ranged from 1 (Ru47a) to 16 (Ru126b3), with a mean number of alleles per locus of 6.4. The percentage of polymorphic loci ranged from 34.6% for the populations S4 and S7 to 85.2% for the population NN7, with an average of 96.4% across all cloudberry samples. Nei's average gene diversity values ranged from 0.194 in population S7 to 0.450 in population NN4 (Table 1). The overall gene diversity for all populations was 0.463. The Shannon index was lowest in Spitsbergen population S4 (*I *=* *0.271), and the highest was in the continental Norway population from Kvaloya island NN7 (*I *=* *0.669; Table 1). The overall value of *I*_total_ was 0.937 when all populations were included.

The level of genetic diversity was the lowest in cloudberry populations from Spitsbergen ($\hat{h}\;=\;0.301$; *I *=* *0.522), of moderate level in populations from the Krkonose Mountains ($\hat{h}\;=\;0.432$; *I *=* *0.782) and the highest in main cloudberry localities from Norway ($\hat{h}\;=\;0.456$; *I *=* *0.902).

**Figure 3:**
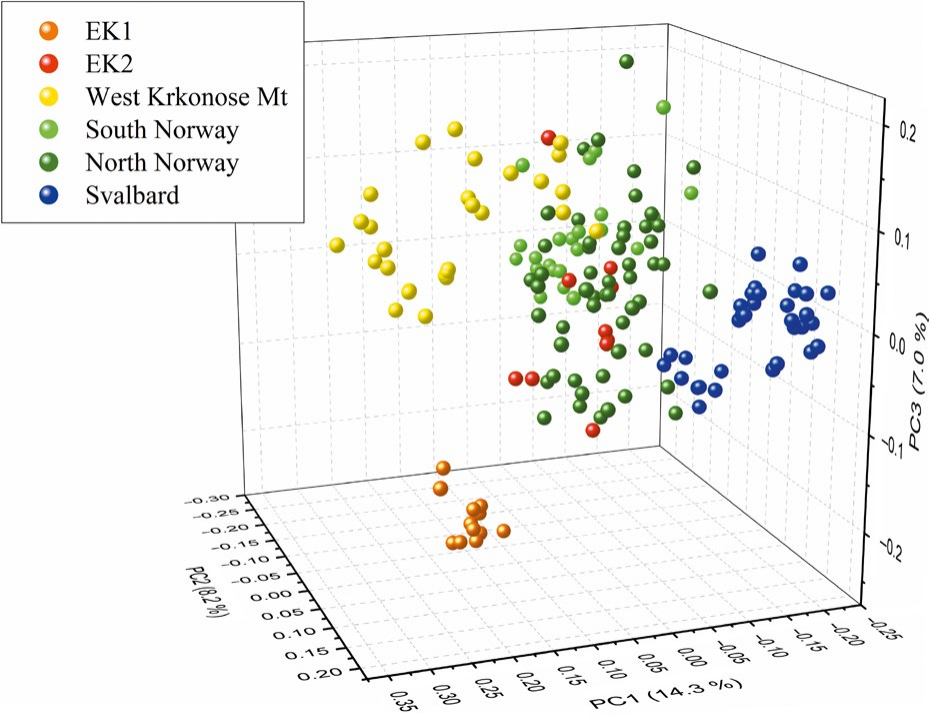
Principal coordinate analysis (PCoA) plot of 162 *Rubus chamaemorus* individuals based on SSR data

Cluster analysis showed three main clusters: two of them include cloudberry samples from CR and from continental Norway, and one cluster formed by cloudberry genotypes from continental Norway and from Spitsbergen. While these main clusters are not supported by bootstrap, many small clusters encompassing the whole or a part of local populations have a bootstrap level higher than 50 (Appendix S2). This indicates population structure within many small local populations. Principal coordinate analysis (PCoA) based on genetic distance between samples indicated differentiation between populations EK (East Krkonose Mountains) and WK (West Krkonose Mountains) and that both are distant from populations in continental Norway and Spitsbergen with the exception of the EK2 population from Certova louka. Cloudberry populations from Spitsbergen are also partly differentiated from continental Norwegian populations (Figure 3). The first three axes represented 30% of total variation.

The genetic structure was then evaluated using Bayesian analyses as implemented by the Structure software. As a shallow level of population structure was supposed, a locprior model (Hubisz et al., 2009) was used. According to the Δ*K* value, six clusters (K1–K6) were identified among *Rubus chamaemorus* populations (see Appendix S3). The mean value of α was 1.689, indicating that most of the cloudberry genotypes were genetically admixed (Falush et al., 2003). The value of *r* was .229 which means that localities were of a high importance for the population structure. Based on the proportion of membership of each population in each of the six clusters (Appendix S6), cluster K1 is typical for Norwegian populations and cluster K2 consisted exclusively of populations EK2 (88%) and SN1 (96%) and in a lower percentage populations SN3 (45%), SN4 (26%), and NN11 (23%). Cluster K3 included only cloudberry populations from Western Krkonose Mountains and partly population EK2 (8%). Cluster K4 consisted of cloudberry populations mainly from Spitsbergen and in a lower percentage populations WK1 (15%), WK4 (23%), SN2 (17%), and NN12 (20%). Cluster K5 included solely the population EK1 (100%) and a part of the population NN10 (26%). Cluster K6 comprised a mixture of cloudberry populations from the north part of continental Norway—NN1 (8%), NN2 (5%), NN3 (9%), NN4 (5%), NN5 (36%), NN6 (1%), NN7 (78%), NN8 (93%), NN9 (73%), NN10 (61%), NN11 (72%), NN12 (42%), Spitsbergen: S1 (91%), S2 (52%)—and two populations from CR—WK1 (21%) and WK4 (7%). The results of the population analysis are represented in Figure 2a. An expected heterozygosity between individuals within the same cluster ranged from 0.275 (K4) to 0.456 (K1), with an average of 0.370. *F*_ST_ values ranged from 0.151 (K1) to 0.555 (K5). The highest values of *F*_ST_ were found in the clusters K3 (0.426) and K5 (0.555) which consisted of cloudberry populations from the Czech Republic and K4 (0.491) which consisted of the populations from Spitsbergen. Figure 2b represents the cluster analysis of all cloudberry genotypes in the 31 populations with *K *=* *6 and shows admixtures among populations (Figure 2b).

The results of the analysis of molecular variance (AMOVA) indicated that 70.8% of the total variation was attributable to differences among individuals within populations, 17.3% was attributable to differences among populations within groups (populations within countries), and only 11.9% was attributable to differences among groups (CR, continental Norway and Spitsbergen, Table 2). Fixation index of all three levels was moderate (*F*_ST_ = 0.29, 0.20, and 0.12, respectively) but statistically significant (*p *< .01).

**Table 2:** Analysis of molecular variance for microsatellite analysis data of *Rubus chamaemorus* populations

Hierarchical level	*df*	Sum of squares	Variance component	Variation (%)	*F*_ST_	*p*
Among groups (Czech Republic, continental Norway and Spitsbergen)	2	118.06	0.47567	11.87	0.12	< .01
Among populations within group	28	279.418	0.69325	17.29	0.20	< .01
Within populations	293	832.04	2.83973	70.84	0.29	< .01
Total	323	1,229.519	4.00865			

**Figure 4:**
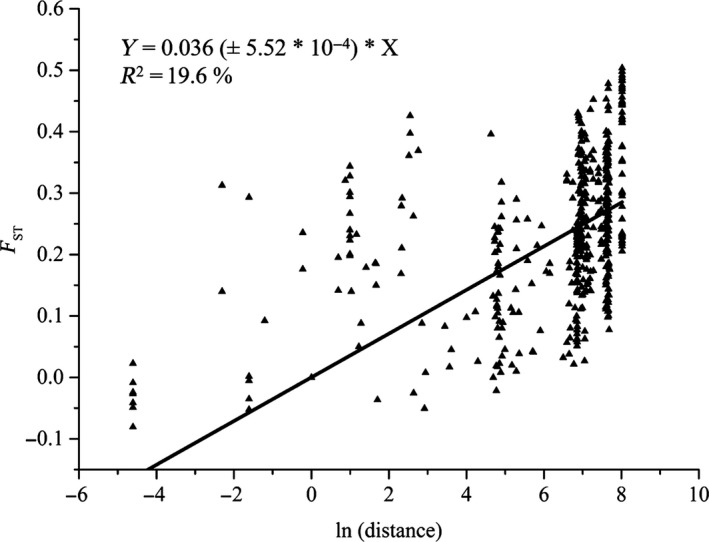
The correlation between pairwise *F*_ST_ values and logarithm of pairwise geographical distance between populations of *Rubus chamaemorus*

The Mantel test revealed a moderate, significant positive relationship between geographical and genetic distances (*r *=0.44; *p *<* *.01) across all the sampled localities, indicating some level of isolation‐by‐distance. If populations where gene flow over a long distance was noticed (EK2, WK1, WK2, NN4, and NN10) were omitted, the value of the correlation coefficient increased (*r *=* *.54; *p *<* *.01). A model of linear regression was selected for the representation of the relationship between geographical and genetic distances (Figure 4).

**Figure 5:**
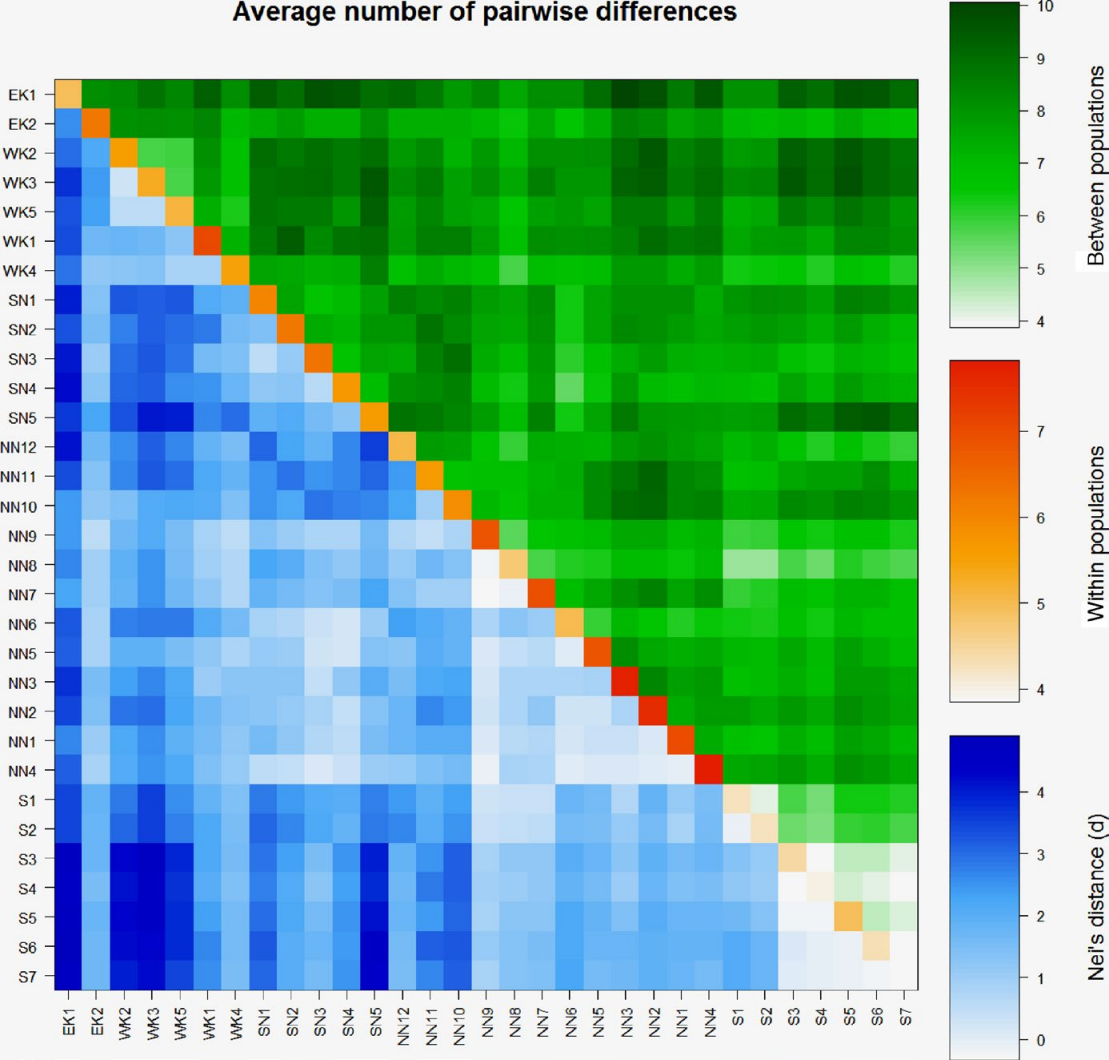
Pairwise difference between *Rubus chamaemorus* populations

The overall *F*_ST_ was high (0.45) but when we considered regions (CR, continental Norway, and Spitsbergen) as populations, *F*_ST_ value was 0.19. The estimated gene flow, *M *= *Nm* was 0.31, and 1.08 when regions were taken as populations. This indicates that some populations are much more differentiated than others and gene flow is generally restricted but occurs between some populations. Almost all pairwise *F*_ST_ values were significant (*p *< .05), ranging from −0.08 to 0.50 (Appendix S4). These results were confirmed by the exact test of population differentiation. Significant differentiation (*df *= 52, 48, 50, resp.; χ^2^ = 419.7; 448.5, 370.4, resp.; *p *< .01 for all three) was found for the pairs of population groups from CR and continental Norway, CR and Spitsbergen, continental Norway and Spitsbergen.Significant differentiation was also found between groups of populations from east and west parts of the Krkonose Mountains (*df* = 52; χ^2^ = 433.8; *p *< .01), groups of populations from the north and south part of continental Norway (*df* = 54; χ^2^ = 285.6; *p *<* *.01), and groups of populations from two localities on Spitsbergen (*df* = 52; χ^2^ = 153.8; *p *<* *.01). Based on the pairwise test of genetic differentiation between all cloudberry populations, the null hypothesis that there is no difference between a pair of populations could not be rejected in 87 pairs of populations (18.7%) (Appendix S5). Pairwise differences and the variation level within populations are presented in Figure 5.

## 4 DISCUSSION

Our analysis of SSR loci indicated that *Rubus chamaemorus* maintained a high overall genetic diversity ($\hat{h}=0.463$) similar to that of *Rubus glaucus* (0.456; Marulanda, López, & Uribe, 2012) and higher than in *Rubus idaeus* from Lithuania *(* $\hat{h}=0.257$; Patamsytë et al., 2005). The highest level of genetic diversity was found in continental Norway ($\hat{h}=0.456$), where populations are more frequently reproduced. The lowest level of genetic diversity was detected in Spitsbergen ($\hat{h}=0.301$) and a moderate level in the Krkonose Mountains, CR ($\hat{h}=0.432$). This high level of genetic diversity is surprising especially in the Krkonose Mountain populations which have been isolated from the main cloudberry growing areas since glacial melting after the end of the Last Glacial period (Engel, Braucher, Traczyk, Laetitia, & Team, 2014; Hultén, 1968). It is known that the level of genetic variability in small isolated populations reduces as a consequence of genetic drift and bottlenecks. The possible explanation for the high diversity of cloudberry populations in this previously glaciated area is, according to Alsos, Engelskjon, Gielly, Taberlet, and Brochman (2005), efficient and broad fronted recolonization from large and diverse populations on the tundra surrounding the glaciers, as well as from more distant populations. *Rubus chamaemorus* occurrence in Central Europe was probably widespread in the areas in the Late‐Glacial periods and has subsequently retreated and survived only on higher altitude peat boggy places with enough water and colder climates (Dostál, 1989).

According to our results, samples from the Krkonose Mountains are three genetically distinct populations (Figures 2 and 3). The first is represented by the whole population EK1 which is well differentiated (*F*_ST_ = 0.32–0.43; *p* < .01) from other CR cloudberry populations. The second true population involves local populations WK2, WK3, WK5 not differentiated from each other (*p* = .17, .39 and .41, resp.; see Appendix S5), but they are differentiated from other CR populations (*F*_ST_ = 0.05–0.43; *p* < .01). The third population involves the whole local population EK2 which is distinct from other CR populations (*F*_ST_ = 0.17–0.32; *p* < .01). So, cloudberry populations in the Krkonose Mountains came most probably from at least three different genetic populations from the last glacial period (Figures 2 and 3). After the glacial period had finished, the three populations may have survived in several localities. Gene flow may have happened from the main *R. chamaemorus* populations in Scandinavia or Russia, as the level of glaciation changed in the Quaternary period (Engel et al., 2014). Therefore, population EK2 clustered within continental Norwegian cloudberry populations SN1, SN3, SN4, NN4, NN11, and NN12. Furthermore, WK1 and WK4 populations slightly differed from other populations in Krkonose Mountains (39% and 32%, respectively) and they are closer to Norwegian populations NN1–NN12 (24% and 9%, resp.) and S1–S7 (15% and 23%, resp.; Figure 2).

Alsos et al. (2005) obtained similar results for *Vaccinium uliginosum* populations and proposed a possibility of recolonization from long‐distance source populations by means of wind, drifting sea ice, drift wood, or birds. Ehrich et al. (2008) studied cloudberry populations through the entire circumpolar area using AFLP markers and found that the source population for Europe is West Siberia with the border in Taimyr, where the highest level of diversity was detected. Vectors of gene flow could be birds living on cloudberry fruits and being able to migrate through long distances, for example, gray lag goose which nests in the Hebrides, Scandinavia, and Russia, winters in the British Isles and has a flight speed and metabolism which seems to permit dispersal of seed between land masses in the North Atlantic (Löve, 1963). Gene flow over long distance could therefore be possible.

*Rubus chamaemorus* largely reproduces itself vegetatively. Few seeds are produced in most areas but in a restricted number of localities, seed is produced regularly in fair quantity, although viability is poor (Taylor, 1971). In the Krkonose Mountains, cloudberry flowers very rarely. The last time cloudberry flowered was in a cool spring in 2005 after a long winter with a good amount of snow (Dvorak, 2005). It seems that the limiting factor for flower development is the humidity of cloudberry habitats (Ehrich et al., 2008). In spite of these difficulties, random hybridization between individuals even from different populations can occur. This is supported by an individual Ru14011 from population EK2 which is a compound of genetic populations from the east (30% involvement in K2 cluster predominant for EK2 population) and from the west part (70% probability of inclusion to cluster K3 encompassing populations WK1–WK5) of the Krkonose Mountains (Figure 2b).

The northernmost populations of cloudberry in Spitsbergen showed the lowest level of diversity. They grouped into two genetic groups: one unique to Spitsbergen populations and one shared between Spitsbergen and continental North Norway populations (Figure 2). If the hypothesis that Spitsbergen was colonized by *Rubus chamaemorus* from the Ural Mountains or from western Siberia (Alsos et al., 2007) is true, then the unique genetic population could be a remnant of the original population from Siberia. Similarly in the Krkonose Mountains, genetic populations represented by clusters K3 and K5 could be remnants of the original gene pool of cloudberry populations. These remnant populations are protected due to the large distance from the current areas of *R. chamaemorus* populations. Fitness of these populations is maintained by intermittent flowering brought about by the occasional opportunity of hybridization between individuals from the same or different populations.

In conclusion, cloudberry populations EK1WK2, WK3, WK5S3, S4, S5, S6, and S7 from the Krkonose Mountains, continental Norway, and Spitsbergen are well differentiated and are likely to represent the original gene pool. In contrast, in populations EK2, WK1, WK4 S1, and S2 there is evidence of regular gene flow and hybridization; therefore, these are not differentiated from populations in continental Norway.

*Rubus chamaemorus* populations from the Krkonose Mountains have a moderate level of genetic variability, which is good for sustainable vitality of such heterogenous populations. They may be less susceptible to climatic fluctuations during changing of climate and other anthropogenic factors. The variability is also important for conservation management of the endangered species in the Czech Republic as it means current management strategies are appropriate along with regular monitoring of populations fitness (Phillips, Asdal, Brehm, Rasmussen, & Maxted, 2016). All local populations of *R. chamaemorus* in the Krkonose Mountains occur within the first zone of the Krkonose National Park, and thus, they have the highest level of protection. On the other hand, the Spitsbergen populations that are restricted to only several fragmented small sites and have a low level of genetic diversity are more threatened. Moreover, as they multiply mostly vegetatively there is a limited gene flow. The diversity of such limited populations is unique and appropriate in situ and ex situ conservation of those populations will be of a high priority. The whole Spitsbergen archipelago is protected; nevertheless, a special attention on *R. chamaemorus* sites would be desirable, especially an increase in monitoring. Conservation of *R. chamaemorus* in the global sense does not mean ensuring the survival of every population, but it is necessary to conserve the widest range of its genetic diversity. It will be necessary to work closely with national experts and conservation managers in the Czech Republic, continental Norway, and Spitsbergen to ensure the range of diversity illustrated here is conserved both in situ and ex situ.

## CONFLICT OF INTEREST

None declared.

## AUTHOR CONTRIBUTIONS

L.L.S., J.P., I.M., and V.H. conceived the ideas and collected samples; L.L.S. conducted genotyping and analyzed data; and all authors contributed to interpretation and writing which was led by L.L.S.

## DATA ACCESSIBILITY

All data are in supporting information, and they will be available at time of publication.
