# Supplementary figures and images for: Genetic differentiation of Rubus chamaemorus populations in the Czech Republic and Norway after the last glacial period
Source: Ecol Evol. 2018 May 2;8(11):5701–11. doi: 10.1002/ece3.4101 (PMC6010844; doi:10.1002/ece3.4101)

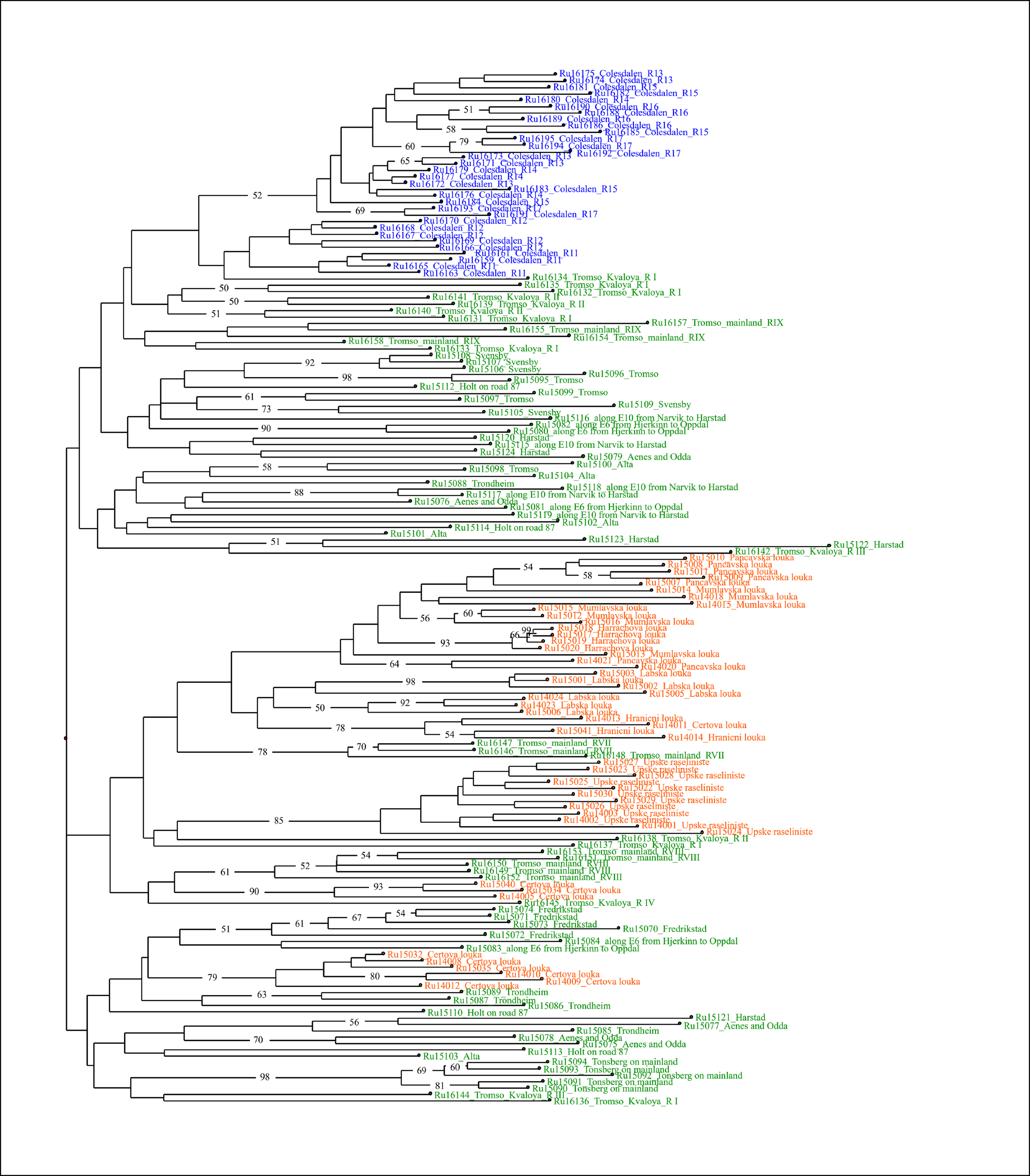

Supplement: Supplementary file 1 [file ECE3-8-5701-s001.png]
