# Supplementary material for: Genetic differentiation of Rubus chamaemorus populations in the Czech Republic and Norway after the last glacial period
Source: Ecol Evol. 2018 May 2;8(11):5701–11. doi: 10.1002/ece3.4101 (PMC6010844; doi:10.1002/ece3.4101)
Supplement: Supplementary file 2 [file ECE3-8-5701-s002.docx]

| **Kriteria for choice of number of groups** | | |  |  |  |
| --- | --- | --- | --- | --- | --- |
| 1. Relative plateau after K in Ln(p) | | |  |  |  |
| 2. Low variation of Ln(p) in K | |  |  |  |  |
| 3. Drop in Ln'(p) after K. | |  |  |  |  |
| 4. Peak in delta(p). |  |  |  |  |  |
| [Evanno et al. (2005) Mol Ecol 14:2611-2620] | | | |  |  |

Supporting information

Additional Supporting Information may be found in the online version of this article:

Table S1 List of all analysed samples.

Table S2 List of microsatellite alleles of 162 cloudberry genotypes.

Table S3 Summary of population structure analysis - computation results serving to find the value of *K*.

Table S4 Matrix of population pairwise values of F_ST_.

Table S5 Matrix of p values of pairwise population differentiation analysis.

Table S6 Proportion of membership of each pre-defined populations in each of the 6 clusters.

Figure S1 Dendrogram of all samples based on genetic distances computed using UNJ dissimilarity coefficients.

Figure S2 Summary of population structure analysis - graphs serving to find the value of *K*.
